# Antiphytoviral Activity of *Satureja montana* L. ssp. *variegata* (Host) P. W. Ball Essential Oil and Phenol Compounds on CMV and TMV 

**DOI:** 10.3390/molecules15106713

**Published:** 2010-09-28

**Authors:** Valerija Dunkić, Nada Bezić, Elma Vuko, Dubravka Cukrov

**Affiliations:** Department of Biology, Faculty of Science, University of Split, Teslina 12, 21000 Split, Croatia; E-Mails: bezic@pmfst.hr (N.B.); elma@pmfst.hr (E.V.); dubravkacukrov@gmail.com (D.C.)

**Keywords:** : carvacrol, CMV, essential oil, *satureja montana* L. ssp. *variegate*, thymol, TMV

## Abstract

The essential oil of *Satureja montana* L. ssp. *variegata* (Host) P. W. Ball (Lamiaceae) was characterized by a high concentration of oxygenated monoterpenes (71.3%), among which carvacrol (19.4%) and thymol (16.6%) were the major compounds. When the essential oil was applied on local hosts *Chenopodium amaranticolor* Coste & Reyn. and *Chenopodium quinoa* Willd. simultaneously with the infecting virus, the number of local lesions on both Tobacco mosaic virus (TMV) and Cucumber mosaic virus (CMV) infected plants was reduced for 29.2% and 24.1%, respectively. When applied individually for each virus, thymol was more effective in reducing CMV infection (33.2%), while carvacrol was more effective in reducing the TMV infection (34.3%). No synergistic effect of both monoterpenes was observed in the antiviral activity of the oil.

## 1. Introduction

Plants in the family Lamiaceae are a very important source of essential oils and other biologically active molecules. Essential oils are variable mixtures, principally of terpenoids, and specifically of monoterpenes and sesquiterpenes, although diterpenes may also be present. Monoterpenes are detected in every essential oil—comprising from as little as 1% to more than 95% of the oil and are usually present as main constituents in oil fractions of *Satureja* plants [1]. The investigated plant, *Satureja montana* L. ssp. *variegata* (Host) P. W. Ball. was collected in Dalmatia, the Mediterranean part of Croatia.

An extensive body of research has demonstrated that essential oils and their main components posses a wide spectrum of biological activities, which may be of great importance in several fields. These compounds are important for the physiological function of growth, ecological function and development [2]. Moreover, they play an important role in the resistance against diseases and insects [3]. The antimicrobial activity of the essential oils constituents has already been fully discussed by many authors [4,5,6]. Besides anti-bacterial properties, essential oils and their components have been shown to exhibit antiviral, antimycotic, antioxygenic, antiparasitic and insecticidal properties [7,8,9,10,11]. The phenol components with hydroxyl groups were found to posses the major antimicrobial activity; carvacrols have anti-inflammatory activity and limonenes are antiviral [12]. It is known that the essential oil of *Salvia fruticosa* has a strong activity against *Herpes simplex* virus [13]. The antiviral activity of savory’s essential oils against HIV has been documented [14]. Nevertheless, there is still little information available about the effects of essential oils on viruses or viral infections in plant systems [8].

This paper reports the results of a study on the effects of *S. montana* essential oil and its major components thymol and carvacrol on the development of local lesions caused by Tobacco mosaic virus and Cucumber mosaic virus. Both phenolic compounds are biologically active—thymol has antiseptic, and carvacrol possesses antifungal properties [15]. Thymol and carvacrol are structurally very similar, having the hydroxyl group at a different location on the phenolic ring. Although among the essential oil constituents phenolic compounds with hydroxyl groups were previously described as antimicrobial agents, our research has shown that they can also be potent antiphytoviral agents.

## 2. Results and Discussion

Essential oil of *Satureja montana* L. ssp. *variegata* (Host) P. W. Ball was obtained by hydrodistillation using a Clevenger-type apparatus and analyzed by GC/FID and GC/MS. The yield of the oil was 2.5% and the oil was yellow in color. Thirty compounds were identified, representing 96.3% of the total oil. The qualitative and quantitative compositions are presented in Table 1, where compounds are listed in order of their elution on the VF-5ms column. The oil was characterized by a high concentration of oxygenated monoterpenes (71.3%) of which carvacrol (19.4%) was the major compound, followed by thymol (16.6%) and linalool (5.9%). Monoterpene hydrocarbons constituted 22.6% of the oil and *γ*-terpinene (6.9%) and *α*-terpinene (4.9%) were the main compounds of this fraction. Sesquiterpenoids comprised 2.4% of the total oil.

Results obtained in our work are comparable to other published studies. The percentage of carvacrol in different samples of *S. montana* varied from 5.3% in some samples reported by Radonić *et al.* [16], 57% in some Italian winter savories [17], up to 84% for some samples collected from the central part of Dalmatia [18], where thymol was a dominant compound (45%). It has been shown that the production of phenolic compounds is stimulated by hot and dry environmental conditions. The essential oils isolated from various species of *Satureja* have biological properties such as antimicrobial [19,20], antioxidant [21], antispasmodic and antidiarrhoeal [22,23].

**Table 1:** Phytochemical composition (%) of essential oil in *Satureja montana* L. ssp. *variegata*.

No.	Component	RI VF-5ms	RI CP Wax 52	Yield %
1.	α-Thujene	924	1035	0.8
2.	α-Pinene	935	1032	0.2
3.	Camphene	947	1059	0.2
4.	1-Octene-3-ol	974	1452	0.8
5.	Myrcene	988	1174	4.0
6.	Linalool oxide	991	1479	0.9
7.	α-Terpinene	1016	1188	4.9
8.	*p*-Cymene	1021	1268	2.7
9.	Limonene	1028	1203	0.8
10.	(*Z*)-β-Ocimene	1032	1218	1.6
11.	γ-Terpinene	1057	1255	6.9
12.	Sabinene hydrate	1065	1474	0.8
13.	Linalool	1097	1553	5.9
14.	*allo-*Ocimene	1128	1351	0.5
15.	Camphor	1143	1499	0.6
16.	Borneol	1165	1719	3.0
17.	Terpinen-4-ol	1174	1611	2.1
18.	α -Terpineol	1186	1646	1.9
19.	Myrtenol	1194	1804	0.8
20.	Nerol	1227	1808	3.0
21.	Thymol methyl ether	1230	1604	4.1
22.	Carvacrol methyl ether	1241	1614	5.5
23.	Geraniol	1249	1857	1.7
24.	Thymol	1290	2198	16.6
25.	Carvacrol	1298	2239	19.4
26.	Neryl acetate	1358	1692	4.2
27.	Aromadendrene	1439	1628	0.4
28.	δ-Cadinene	1522	1773	0.5
29.	Spatulenol	1578	2144	0.8
30.	Caryophyllene oxide	1582	1927	0.7
	Monoterpene hydrocarbons			22.6
	Oxygenated monoterpenes			71.3
	Sesquiterpene hydrocarbons			0.9
	Oxygenated sesquiterpenes			1.5
	Total			96.3

RI = retention indices.

**Figure 1:**
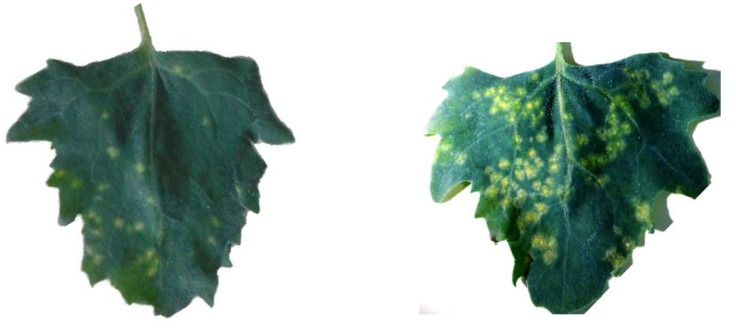
(**a**) Reduction of TMV local lesion number on the carvacrol treated (right) and untreated (left) leaf half of *Chenopodium amaranticolor* Coste & Reyn; (**b**) Reduction of CMV local lesion number on thymol treated (right) and untreated (left) leaf half of *Ch. quinoa* Willd.

A search of the literature dealing with antiphytoviral effects of pure essential oils resulted in only a few articles [8,24]. Essential oil of *Melaleuca alternifolia* was previously reported as an inhibitor of Tobacco mosaic virus [8]. When the oil was applied onto *N. glutinosa* plants as a pre-inoculation spray, the number of local lesions was significantly inhibited. Crude extract and the essential oil of *Plectranthus tenuiflorus* also showed an inhibitory effect against Tobacco necrosis virus, Tobacco mosaic virus and Tomato spotted wilt virus [24]. The essential oil of *Satureja montana* has been proven to have powerful inhibitory effects against several bacterial and fungal pathogens [20], but antiphytoviral activity of the oil has not been tested so far. In our experiment the oil is mixed with the viral inoculum and tested on the local hosts against Tobacco mosaic virus (TMV) and Cucumber mosaic virus (CMV). Thymol and carvacrol, phenolic compounds with hydroxyl groups are the most abundant compounds in the essential oil (Table 1) and the individual antiphytoviral activity of these major oil components was also tested. Our experiments aimed to know whether these monoterpens were responsible for the antiviral activity of the oil and whether there is a synergism in their antiviral effect in the oil. When the oil was applied on local hosts simultaneously with the infecting virus, the number of local lesions was reduced 29.2% for TMV infection and 24.1% for CMV infection (Table 2). When applied individually, thymol and carvacrol reduced the number of local lesions on both CMV- and TMV-infected plants. The inhibitory effect of thymol was stronger in the reduction of CMV infection with the antiviral activity rate of 33.2% (Figure 1, Table 2). A slightly lower efficiency was recorded for TMV infection with a rate of 26.1%. On the other hand, carvacrol was more efficient in reducing the number of local lesions on the TMV infected plants, with an activity rate of 34.3% (Figure 1, Table 2). The percentage of inhibition of CMV infection with carvacrol was 28.3% (Table 2). Comparing the percentages of inhibition, it is obvious that there is no synergistic action of thymol and carvacrol in the antiviral activity of the oil.

The use of natural resources from plant species in the treatment of plant viral diseases is an extensively explored area, and this paper provides some new information about so far unexplored antiphytoviral activity of *S. montana* L. ssp. *variegata* essential oil. It is also confirmed that phenolic compounds with the hydroxyl group are not only antimicrobial agents, but also antiphytoviral agents. However, some other questions about the antiphytoviral mechanisms of essential oil and its components should be solved.

**Table 2:** Effect of essential oil/thymol/carvacrol on TMV and CMV infectivity.

	*TMV*			*CMV*		
	Mean of L.L ±SEM (treated)	Mean of L.L ±SEM (control)	% of inhibition	Mean of L.L ±SEM (treated)	Mean of L.L ±SEM (control)	% of inhibition
Essential oil	15.0 ± 0.8*	21.2 ± 0.8	29.2	24.9 ± 1.0*	32.8 ± 1.0	24.1
Thymol	29.8 ± 2.4*	40.3 ± 2.3	26.1	22.1 ± 2.5*	33.1 ± 2.5	33.2
Carvacrol	15.3 ± 1.1*	23.3 ± 1.4	34.3	18.5 ± 1.1*	25.8 ± 1.2	28.3

Mean of L.L = the mean number of local lesions; SEM= Standard Error Mean; *Significance reduction in disease compared with control (p ≤ 0.05).

## 3. Experimental

### 3.1. Plant Material for Essential Oil Analysis

Plant material of *Satureja montana* L. ssp. *variegata* (Host) P. W. Ball was collected from the Jezera (Murter Island near the city of Šibenik) in the summer (August) 2009. Voucher specimens are deposited in herbarium at the Department of Biology, Faculty of Science, University of Split, Croatia [No. FS 2009: 11, a].

### 3.2. Plant Hosts for Antiphytoviral Studies

Seeds of *Chenopodium amaranticolor* Coste & Reyn. and *Ch. quinoa* Willd. were sown in trays containing Klasmann universal compost and maintained in a greenhouse (23 ºC, 16:8 h light/dark cycle) with watering as required. When the seedlings were large enough to handle they were potted individually into 10 cm plastic pots containing fresh compost. Plants were grown in a greenhouse under same conditions. Experimental plants were selected three weeks after sowing, when they had four true leaves. Care was taken to ensure that the experimental plants were as uniform in size as possible.

### 3.3. Isolation and GC-MS Analysis of Essential Oils

Aerial parts of plants were performed in a shady place at room temperature for 10 days. Plant tops during flowering were used for the analysis of essential oil composition. Dried aerial parts of plant material (100 g) were subjected to hydrodistillation for 3 h in Clavenger type apparatus. The obtained essential oil was dried over anhydrous sodium sulphate and 1 µL was used for GC/FID and GC/MS measurements.

GC/FID analyses were performed on a Varian 3900 gas chromatograph equipped with a flame ionization detector (FID) and two columns: a VF-5ms capillary column (30 m × 0.25 mm, film thickness 0.25 μm) with 5%-phenyl-95%-dimethylpolysiloxane as the stationary phase and a CP-Wax 52 CB (30 m × 0.25 mm, film thickness 0.25 μm) with polyethylene glycol as the stationary phase. Hydrogen was used as the carrier gas flow rate 1.2 mL/min; the injection volume 1 μL with a 1:10 split ratio. GC oven temperature was kept at 50 °C for 5 min, and programmed to 250 °C at a rate of 5 °C /min.

GC/MS analyses were carried out on a Varian Saturn 2000 system equipped with a VF-5ms and CP-Wax 52 CB capillary columns; with similar temperature programmed as in GC, transfer line temperature 250 °C, carrier gas helium with a linear velocity of 31.5 cm/s, split ratio 1:60, ionization energy 70 eV, ion source temperature 280 °C, mass range 40—600 mass units.

The individual peaks were identified by comparison of their retention indices as *n*-alkanes to those of authentic samples and literature [25], as well as by comparing their mass spectra with the Wiley 6.0 library (Wiley, New York) and NIST/02 mass spectral database. The percentage composition of the samples was computed from the GC peak areas using the normalization method.

### 3.4. Solutions of Essential Oil*, *Thymol and Carvacrol

Soluble solution of essential oil in inoculation buffer was prepared for testing the antiphytoviral effect. To overcome insolubility of the oil in buffer, 100 μL of oil was mixed with 0.1 mL of Tween 80 and subsequently made up to 150 mL with prepared viral inoculum. Thymol (Kemika) was dissolved in dimethyl sulfoxide (DMSO) to give the concentration 1 mol/L. The stock solution was stored at 4 °C. The final concentration of the thymol in viral inoculum was adjusted to 1 mmol/L. Carvacrol (Roth) was added directly in viral inoculum to give the final concentration of 4.2 mmol/L.

### 3.5. Viral Inoculum

Viral inoculum was prepared from leaf tissue obtained from *Nicotiana tabaccum* L. cv. Samsun and *N. megalosiphon* Van Heurck & Muell.Arg. plants previously infected with Tobacco mosaic virus and Cucumber mosaic virus, respectively. Leaves of systemically infected leaf material (3–5) were ground with inoculation buffer (15 mL, 0.06 mol/L phosphate buffer, pH = 7.0) in a mortar. Sap extract was diluted with the same inoculation buffer to give a suitable number of discrete local lesions on test plants. The inoculum prepared in this way was used to inoculate local hosts *Ch. amaranticolor* and *Ch. quinoa*.

### 3.6. Application to the Local Host Plants

Antiphytoviral activity of the essential oil and its constituents against TMV and CMV was determined by the half-leaf method. One half of the leaf was inoculated with the virus inoculum containing essential oil, thymol or carvacrol. The opposite half of the same leaf was inoculated with the viral inoculum (tween 80 was added in experiment with essential oil, and DMSO was added in experiment with thymol). All treatments were repeated for three times on plants selected for uniformity and grown in a greenhouse (23 °C; 16:8 h light/dark cycle). Local lesions were counted 6 days post inoculation (p.i.) and the inhibition percentage was calculated by comparing the number of viral lesions on the two leaf halves according to the formula:

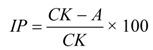

where IP = antiviral inhibition in %, CK = average number of viral lesions on the control leaf half; A = average number of viral lesions on the essential oil/thymol/carvacrol treated leaf half.

### 3.7. Statistical Analysis

The significance of difference between mean value for treatment and control was estimated statistically using one tailed student T-test.

## 4. Conclusions

GC/FID and GC/MS analyses of *S. montana* L. ssp. *variegata* essential oil identified thirty compounds. The oxygenated monoterpenes, thymol and carvacrol were the major compounds. Our research confirmed that *S. montana* essential oil, as well as its main phenol components, could be antiphytoviral agents. A statistically significant reduction of local lesion number was detected when the oil/thymol/carvacrol was applied on local hosts simultaneously with infecting virus. Comparing the percentages of inhibition, it is noted that carvacrol was more effective in reducing TMV infection, while thymol was stronger inhibitor of CMV infection. The antiphytoviral effect of the oil was slightly lower compared with individual phenol components and indicated that there is no synergism in their antiviral activity in the oil. Results in our work may help to improve the method of application and possible utilization of these compounds in the control of plant virus diseases.
